# Supplementary material for: Clinicopathological and molecular subtypes of breast cancer in the Eastern Cape, South Africa: A two-year retrospective study
Source: PLoS One. 2025 Jul 21;20(7):e0325387. doi: 10.1371/journal.pone.0325387 (PMC12279119; doi:10.1371/journal.pone.0325387)
Supplement: Attachment — Submitted filename: Rebuttal Letter - Dr Hayden Kretzmann.docx [file pone.0325387.s003.docx]

**REBUTTAL LETTER:**

**Reviewer #1:**

This is a well-conducted, timely, and comprehensive retrospective analysis highlighting the clinicopathological and molecular profile of breast cancer patients from an underrepresented region in South Africa. The study fills an important data gap and presents detailed insights that are regionally and globally relevant, especially for low- and middle-income country (LMIC) settings. The manuscript is overall well-structured and thorough. However, there are areas that would benefit from clarification, deeper discussion, and minor editing for consistency and precision.

**Response:** Thank you for your comment.

The molecular classification based on IHC is appropriate, but combining Luminal B (HER2– and HER2+) may oversimplify biologically distinct entities. Since numbers for HER2+ tumours are small, this is understandable for analysis, but it should be more explicitly discussed as a limitation.

**Response:** Thank you for the insight. We have added this under the limitations section.

A table summarizing significant associations (e.g., subtype vs grade, stage, delay, etc.) in a concise multivariate format would greatly enhance readability.

The finding that only 4.5% underwent breast conservation surgery is critical. The authors should comment more explicitly on the infrastructure, surgical expertise, and patient-related factors that influence this low rate.
The data on non-surgical management (41.6% did not undergo surgery) needs elaboration—was this due to stage IV disease, resource limitation, or patient refusal?

**Response:** Thank you for your comment. We have made the necessary additions.

Title: Consider revising to highlight the focus on molecular subtypes:
“Clinicopathological and Molecular Subtypes of Breast Cancer in Eastern Cape, South Africa: A Two-Year Retrospective Study”.

**Response:** Thanks for your comment. We have revised accordingly.

Abstract: Very informative. However, avoid redundancy (e.g., phrase “BC molecular subtypes of BC”).

**Response:** Thanks for your comment. We have made corrections.

Tables: Tables are well-prepared but very dense. Some tables can be moved to supplementary material if needed.

**Response:** Thank you for your comment. We have summarised the significant associations of the breast cancer molecular subtypes with the clinicopathological characteristics and created Table 5 for ease of readability, as requested. The original table 5, table 6 and table 7 have been moved to Table S1, Table S2 and Table S3, respectively, to the supplementary material/ supporting information section.

Grammar and Style:
Some repetition (e.g., “clinicopathological and molecular profile of BC”) should be avoided.
Ensure consistency in use of acronyms (e.g., always define TNBC, HER2 at first mention in each section).

**Response:** Thanks for your comment. We have corrected all redundancy in the paper.

**Reviewer #2:**

Dear authors, thank you for this interesting manuscript, here are my comments for more clarity:

#Missing Data Handling: The limitations section notes missing demographic and clinical data due to the retrospective nature, but methods do not specify how this was addressed (e.g., exclusion, imputation). Clarification is essential for transparency.

**Response:** Thanks for your comment. We have provide additional information in the methods section.

#Consider including STROBE checklist as supporting information for transparency.

**Response:** Thanks for your comment. We have added a STROBE checklist as suggested.

\# Standardize table formatting (e.g., alignment, decimal places, abbreviations like "NOS" and "TNBC" should be defined in footnotes).

**Response:** Thanks for your comment. We have corrected all these in the paper.

#Correct minor errors (e.g., "timeous referral" → "timely referral"; "HER2-enriched in 18.6% and 5.5% of cases" → "HER2-enriched in 5.5% of cases").
\# Ensure consistent terminology (e.g., "LMIC" vs. "LMC"; define all abbreviations at first mention).

**Response:** Thank you for your comments. We have made corrections as suggested.
